# Influence of Immune Myeloid Cells on the Extracellular Matrix During Cancer Metastasis

**DOI:** 10.1007/s12307-016-0181-6

**Published:** 2016-03-09

**Authors:** David Jiang, Su Yin Lim

**Affiliations:** Department of Oncology, CRUK/MRC Oxford Institute for Radiation Oncology, University of Oxford, Old Road Campus Research Building, Roosevelt Drive, Oxford, OX3 7LJ UK

**Keywords:** Tumor microenvironment, Myeloid cells, Cancer metastasis, Metalloproteinases, Cancer therapy, Immune cells

## Abstract

The extracellular matrix (ECM) is one of the most important components within the tumor microenvironment that supports cancer development and metastasis. Under normal physiological conditions, the ECM is a tightly regulated network providing structural and biochemical support. However, the ECM becomes highly disorganized during neoplastic progression and consequently, stimulates cancer cell transformation, growth and spread. Cancer development and progression is also known to greatly benefit from the support of immune myeloid cells, which have multiple pro-tumorigenic functions including promoting tumor growth, migration and invasion, stimulating angiogenesis and suppressing anti-tumor responses. An increasing number of studies have shown that myeloid cells alter the ECM to support metastatic cancer progression and in turn, the ECM can influence the function of infiltrating myeloid cells. However, the exact nature of this relationship, such as the mechanisms employed and their molecular targets remains unclear. This review discusses evidence for the reciprocal dependence of myeloid cells and the tumor ECM for efficient tumor development and explores potential mechanisms involved in these interactions. A better understanding of this relationship has exciting implications for the development of new therapeutic treatments for metastatic cancer.

## Introduction

Cancer cells are dependent on a wide variety of supporting factors for their progression. In their landmark reviews, Hanahan and Weinberg proposed ten key hallmarks that are required by cancer cells for successful tumor development and metastasis [1, 2]. Whilst the acquisition of oncogenes, the inhibition of tumor suppressor genes and other intrinsic genetic factors are key components of these hallmarks, it is becoming increasingly clear that tumor malignancy is also dependent on extrinsic factors involving interactions with stromal cells within the tumor microenvironment. Although these extrinsic factors are much less well understood, and may have little potential in inducing initial cancer cell transformation compared to intrinsic factors, the support from surrounding stromal cells have been shown to be indispensable for efficient metastatic progression [3].

The dependence of cancer cells on extrinsic factors for malignant progression was, and still is, an extremely exciting realization. Targeting genetic changes involved in initial cancer cell transformation is difficult and may not be a feasible therapeutic option. However, inhibiting tumor growth, and more importantly, suppressing the metastatic process may be attainable by targeting cancer-stromal cell interactions. Therapeutic strategies aimed at inhibiting and/or decreasing cancer metastasis is urgently required. As shown in studies conducted by Hayat et al. the survival rates of patients with new cancer diagnoses depend greatly on the initial cancer stage, with far lower survival rates associated with advanced metastatic disease [4]. Furthermore, a significant proportion of patients with new cancer diagnoses will advance to metastatic disease within 2 months and indeed, many patients already have detectable metastatic spread at the time of diagnosis. This only reinforces the need for selective therapeutic strategies to limit the development and progression of metastatic disease.

Amongst the stromal cells in the tumor microenvironment, immune myeloid cells have been reported to play key roles in the metastatic process. In this review, we first highlight the importance of myeloid cells in metastatic progression. Additionally, we discuss the role of the extracellular matrix (ECM), highlighting key proteins within this structure that play predominant functions in metastatic progression. Finally, we conclude by discussing the interaction and interdependence of myeloid cells and the ECM within the tumor microenvironment, and the potential of targeting myeloid cell-ECM interactions for therapy.

## The Metastatic Cascade and the Involvement of Myeloid Cells

### The Metastatic Cascade

Metastasis is a multistep process, greatly influenced by interactions between tumor cells and other cells and components of the tumor microenvironment [5, 6]. After initial cancer cell transformation and tumor growth, cancer cells may leave the primary tumor site as a result of changes within the ECM and intravasate into the bloodstream or lymphatics. If the cancer cells survive in the circulation, they then arrest within distant organs (seeding) due to capillary size restriction and binding to coagulation factors. After extravasating into the tissues of the target organ, cancer cells will subsequently settle at pre-metastatic niches which provide the necessary factors to support the development of macrometastases and eventual colonization of the organ. Each step of the metastatic cascade is highly inefficient, reflecting the survival challenges cancer cells must overcome in order to establish distant metastases [7]. For example, many primary tumors succeed in shedding cancer cells into the circulation but only a few are able to avoid destruction by immune cells or death by anoikis [8] and indeed, an important measure of metastatic potential is the ability of cancer cells to survive outside the tumor microenvironment.

### Immune Myeloid Cells in Cancer

Numerous studies now point to immune cells being one of the most influential factors in stimulating the malignant potential of tumor cells; those of the myeloid lineage appear to be amongst the most important contributors. Immune myeloid cells consist of three main groups of terminally differentiated cells; granulocytes, monocytes/macrophages and dendritic cells, with the granulocytes being further divided into neutrophils, eosinophils, mast cells and basophils. Granulocytes are responsible for releasing a variety of cytokines to mediate inflammatory processes in response to infection and injury. Neutrophils, in particular, have a central role in being able to phagocytose invading pathogen [9]. Similar to neutrophils, monocytes are recruited to sites of inflammation and differentiate into macrophages in order to phagocytose pathogens, apoptotic cells and also mediate tissue repair [10]. Dendritic cells, as prototypic antigen presenting cells that process foreign antigens, bridge the gap between innate and adaptive immunity by priming the humoral and adaptive immune responses [11]. All these cell types are indispensable for the full functioning of an immune system.

It is becoming clear that within the tumor microenvironment, the normal functions of myeloid cells are changed [12]. Numerous studies have shown that the tumor microenvironment preferentially polarizes myeloid cells into tumor-supporting phenotypes such as the myeloid-derived suppressor cells (MDSCs) to suppress cytotoxic immune responses and promote cancer progression. Abnormal dendritic cell myelopoiesis and abnormal dendritic cell function within the tumor microenvironment also contribute to the inability to recruit potent cytotoxic immune responses against tumors [13, 14]. Macrophages are also known to exhibit cancer-promoting activity, leading to their designation as tumor-associated macrophages (TAMs) [15, 16]. Studies have demonstrated that TAMs shift away from the M1 “activated” phenotype which is highly tumoricidal, towards the M2 “alternatively activated” phenotype thought to be important for immunomodulation, tissue repair and supportive of cancer progression [17]. TAMs do not produce IL-12 and consequently, do not recruit the cytotoxic responses of natural killer and T_H_1 cells. Instead, they produce IL-10, driving the polarization of T_H_2 responses, which instead support cancer progression. TAMs also produce CCL22, which recruits immunosuppressive T regulatory cells that inhibit T cell activation [18]. In addition to TAMs, tumor-associated neutrophils (TANs) have also been described [19]. Fridlender et al. showed that TGFβ signaling within the tumor was responsible for inducing a population of N2 “pro-tumor” TANs instead of N1 “anti-tumor” TANs [20]. N2 TANs produce low levels of inflammatory cytokines, do not potently activate cytotoxic CD8^+^ T cells and also produce large amounts of arginase, which inhibits T cell responses [21]. Again, these results demonstrate the ability of tumors to abrogate anti-tumor responses by altering myeloid cell functions to create a supportive environment for growth. Altogether, these findings point to supportive roles for most myeloid cells in tumor growth and metastasis within the tumor microenvironment [22, 23].

### Immune Myeloid Cells during Cancer Metastasis

Myeloid cells have been shown to favor many steps of the metastatic cascade, including cancer cell intravasation, extravasation from the blood, and arrest in distant organs [15, 24, 25]. During intravasation, cancer cells associate with TAMs via EGF-CSF1 signaling to facilitate this process [26]. Once in the circulation, cancer cells are subjected to destruction from the shear force of the circulation as well as from patrolling immune cells. In response, tumor cells and myeloid cells produce osteopontin which protects tumor cells from apoptosis [27, 28]. In addition, cancer cells are able to circumvent eradication by associating with fibrin clots and blood platelets, which helps protect against shear stress and shields cancer cells from immune targeting. Formation of these aggregates also facilitates trapping of cancer cells in small vessels and capillaries, leading to their arrest and adhesion to the endothelium [5]. Circulating cancer cells employ similar mechanisms as immune cells, involving selectins, integrins and cell adhesion molecules, for adhesion to the vascular endothelium. Following adhesion, cancer cells extravasate out of the circulation, likely with the aid of TAMs [29] in a manner similar to intravasation.

Myeloid cells are also able to support metastatic outgrowth once secondary implantation has taken place. Stromal and cancer cells of the tumor microenvironment upregulate a wide range of chemokines and other chemoattractants including CCL2, CCL5, CXCL12, M-CSF, VEGF and TGFβ in order to recruit myeloid cells [24, 30–32]. In addition, hypoxia resulting from ischemic areas of the tumor can induce chemoattractants that stimulate myeloid cell recruitment [33]. Once recruited into the tumor microenvironment, myeloid cells upregulate a number of different growth factors such as EGF, FGF2 and platelet-derived growth factor to promote cancer growth and progression [34, 35]. Aside from promoting myeloid cell recruitment, hypoxia is also important in enhancing growth factor expression by myeloid cells [35].

Although recruitment of myeloid cells to actively growing tumors is undoubtedly important to support further progression, a number of studies have demonstrated that myeloid cell recruitment to metastatic sites is equally important for cancer cell implantation. This early recruitment allows myeloid cells to create an environment optimized for rapid development of macrometastases, also known as the “pre-metastatic niche” [36]. Myeloid cells were found to aggregate at sites of liver metastases and the prevention of their recruitment hindered metastasis foci expansion, decreased tumor burden and prolonged the survival of tumor bearing mice [37, 38]. Kaplan et al. showed that VEGFR1^+^ myeloid cells were recruited to pre-metastatic lung sites before metastatic spread and the removal of these myeloid cells prevented cluster formation and tumor metastasis [39].

Numerous studies have aimed to elucidate the mechanisms employed by myeloid cells to create a pre-metastatic niche once they have been recruited to secondary sites. One mechanism is the stimulation of angiogenesis, mediated through the production of numerous factors including VEGF, basic FGF, TNFα and numerous MMPs known to support angiogenesis [33, 40]. In addition, it is thought that MDSCs are able to differentiate into endothelial cells and directly contribute to the formation of vascular networks. Another key mechanism in the establishment of the pre-metastatic niche is the suppression of anti-tumor responses, which will prevent the destruction of newly implanted cancer cells before they have a chance to expand. As mentioned previously, the phenotypes of myeloid cells within the tumor are altered to be more permissive and even supportive of cancer progression. In addition to osteopontin expressed by tumor cells, MDSCs within the tumor microenvironment are also capable of producing osteopontin [27]. The upregulation of osteopontin increases the immunosuppressive activity of MDSCs and increases recruitment of T regulatory cells, thus creating an immunosuppressive microenvironment to support metastatic cancer progression. Also, MDSCs are known to generate oxidative stress in the tumor microenvironment through the production of reactive oxygen species, resulting in impaired T cell activation and function [12]. Finally, a more recently reported role is the ability of myeloid cells to remodel various components of the tissue microenvironment to assist further cancer development; this is discussed in more detail in subsequent sections of the review. Consistent with all these findings, Bingle et al. reported that myeloid cell density in tumors correlated with poor prognosis and that metastatic cancer progression is suppressed in the absence of myeloid cells, strongly suggesting that survival of patients with metastatic disease would be significantly improved by preventing myeloid cell recruitment to tumors [41].

## The Extracellular Matrix and Its Role in the Tumor Microenvironment

**Fig. 1:**
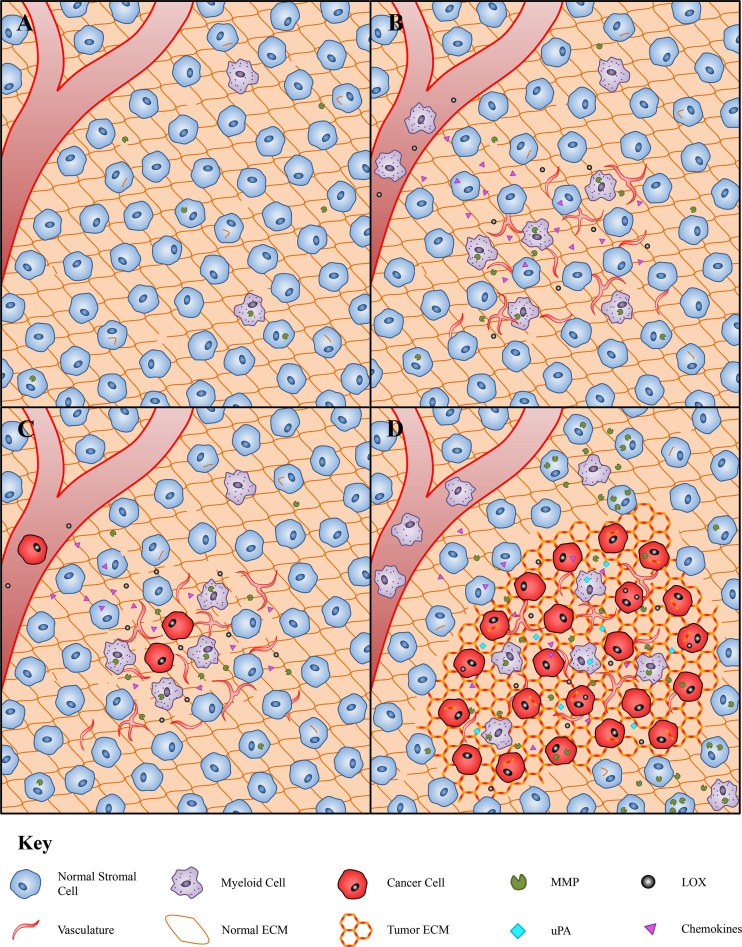
The coordination of myeloid cells and the ECM during cancer progression and metastasis. **a** In the normal tissue environment, physiological levels of ECM regulating enzymes maintain ECM biodynamics to support normal tissue function. In this environment, normal stromal cells comprise a wide variety of cell types including fibroblasts, pericytes and immune cells. **b** During cancer progression, ECM regulating enzymes such as MMPs and LOX are upregulated by cancer cells and aggregate at the pre-metastatic niche where they remodel the ECM. Chemokines produced locally at the pre-metastatic site together with MMPs and LOX promote the recruitment of myeloid cells. The recruited myeloid cells produce angiogenic molecules to establish a rich vascular network. **c** Cancer cells spread from the primary tumor and implant at the pre-metastatic niche. The supply from the rich vascular network and pro-tumorigenic factors produced by myeloid cells stimulate metastatic outgrowth. Upregulated expression and activity of ECM regulating enzymes also contribute to altered ECM and tumor growth. **d** Myeloid cells produce growth factors and angiogenic molecules to ensure continual tumor expansion. ECM regulating enzymes including MMPs, LOX and uPA are upregulated by cancer and myeloid cells, resulting in altered and remodeled tumor ECM that is highly supportive of tumor expansion

The ECM, consisting of a complex network of proteins, glycoproteins, proteoglycans and polysaccharides, is a critical part of the normal tissue environment that provides structural and biochemical support to surrounding cells [42]. Under normal physiological conditions, the components of the ECM undergo a constant cycle of synthesis, deposition, remodeling and degradation, all of which contribute to ECM stiffness, elasticity and function. Deposition of new ECM components is mediated largely by fibroblasts, whereas ECM remodeling and degradation is regulated by a variety of proteolytic enzymes (Fig. 1a) [43]. Due to their dynamic nature and biochemical diversity, the ECM can regulate almost all aspects of cellular behavior and communication through its interaction with cell surface receptors such as integrins [44]. Overall, the reciprocal interaction between the ECM and cells of the surrounding stroma is essential to allow rapid adaptation of tissues to environmental stimuli. Importantly, this makes the alteration of ECM components and biodynamics an effective way to regulate cellular behavior and functions.

The ECM is a significant player in metastatic cancer progression [45, 46] and the dependence of ECM support for both early tumor development and late-stage malignant progression has been demonstrated in several studies [47, 48]. Compared to normal ECM, the ECM within the tumor microenvironment is highly transformed, disorganized and deregulated. Alterations in the composition of the tumor ECM are attributed to overexpression of certain ECM components and their receptors by cancer cells and stromal cells including endothelial cells, fibroblasts and immune cells [49, 50]. ECM stiffness and architectural properties are also altered due to the overexpression of ECM remodeling enzymes such as the lysyl oxidase (LOX) family of enzymes [51]. These biochemical and structural changes in the ECM can influence cell behavior and interactions, which may lead to malignant transformation and metastatic spread (Fig. 1b-d) [52, 53].

Certain ECM components can facilitate the metastatic process by stimulating cell adhesion, attachment and motility [42, 54]. For example, in exposed patches of the basement membrane, laminins promote the attachment of metastatic cancer cells to collagen IV, in order to mediate their arrest and the establishment of early metastatic colonies [55, 56]. Culture of cancer cells on collagen IV/laminin matrices also increased the migration rates of several cancer cell lines [57]. In particular, Koshikawa et al. demonstrated that cancer cells in the presence of laminin-5 upregulated MMP14 expression, which correlated with cancer cell migration [58]. The interaction between cancer cells and abnormal collagen scaffolds at the invasion fronts of tumors has also been shown to promote cancer cell invasion [59–61]. Changes in the stiffness and elasticity of the ECM will also have significant effects since they are detected by surrounding cells [62, 63]. For example, distortion of cellular shape and orientation affect the spatial distribution of integrins expressed by surrounding cells and consequently, affects integrin-mediated signaling [44]. In support of this, Levental et al. demonstrated that collagen cross-linking and the resultant increase in ECM stiffness initiated PI3 kinase signaling cascade to promote oncogenic transformation [64]. In addition, Paszek et al. found that increased collagen deposition and increased ECM stiffness upregulated integrin signaling, which activated focal adhesion kinases and consequently, promoted cancer cell survival, growth and migration [65, 66].

After cancer cell implantation (Fig. 1c), increased ECM deposition within the tumor microenvironment and in the pre-metastatic niche (Fig. 1b) may promote cancer cell survival through the prevention of anoikis. Apoptosis through the anoikis signaling pathway is one of the many challenges cancer cells must overcome during the metastatic process [8]. These signaling pathways are prevented by successful adherence to the ECM, thus increased ECM deposition is likely to aid cancer cell survival. In support of this, growing pancreatic cancer cells on collagen IV matrices has been reported to confer apoptosis resistance, as well as stimulating their growth and migration [67].

Changes in the ECM can also affect other aspects of the metastatic process. For example, laminin is a major constituent of the basement membrane [68], which is essential in supporting the endothelium of blood vessels [69, 70], and has been shown to indirectly support angiogenesis by promoting endothelial cell survival and function [71, 72]. The development of new vasculature also promotes cancer cell metastasis by providing additional routes by which cancer cells can leave the tumor [73]. This is reflected in studies which found that tumor vascularization correlated with metastasis incidence and reduced patient survival in a range of cancers [74, 75].

Collectively, these studies indicate that abnormal ECM composition and dynamics within the tumor microenvironment are essential for cancer development, by accelerating the growth of primary tumors, their spread to distant sites and eventually, the growth of new metastatic colonies. In the following sections, we review several key ECM regulators and discuss how their alteration and deregulation can contribute to cancer malignancy.

### The Matrix Metalloproteinases (MMPs)

A large number of enzymes are responsible for regulating ECM biodynamics and their known roles in cancer progression are summarized in Table 1. Matrix metalloproteinases (MMPs) represent the most abundant ECM regulator within the tissue microenvironment [53, 76]. MMPs cleave ECM components at specific sites in order to regulate ECM availability and biodynamics. MMPs also indirectly regulate cellular behavior by exposing cryptic sites within ECM components that have separate biological functions, and by releasing important signaling molecules stored within the ECM network [77, 78]. Several mechanisms mediate MMP function, including transcriptional regulation, production as inactive precursors, selective distribution and the action of endogenous MMP activators and inhibitors within tissues. However, during malignant progression, MMP activity becomes deregulated, which contributes toward the disruption of normal tissue ECM, and also the abnormal regulation of several signaling pathways [79, 80].

**Table 1:** Key ECM regulating enzymes and their roles in metastatic cancer progression

**ECM regulating enzyme**	**Roles/effects in metastatic cancer progression**	**References**
**MMPs**		
MMP1	Increased expression associated with increased cellular susceptibility for tumorigenesis. After initial tumor development, MMP1 is likely to support cancer cell invasion and the formation of distant metastases.	[93, 154–157]
MMP2	Important roles in supporting tumor angiogenesis through the promotion of epithelial cell migration. Increased expression has also been identified as a marker for enhanced tumor progression and poor patient outcome.	[94, 100, 158–160]
MMP3	Important role in supporting carcinogenesis and initial tumor development. Increased expression is also associated with increased invasive and malignant potential.	[97, 98, 104, 159, 161]
MMP7	Responsible for mediating myeloid cell recruitment to tumors, as well as protecting tumor cells from apoptosis and chemotherapy. Expression is associated with poor outcome in patients.	[139, 162–165]
MMP8	Demonstrated to have protective roles by impairing cancer progression, including inhibition of cancer cell transformation and metastatic spread.	[103, 166, 167]
MMP9	Important roles in tumor angiogenesis and vasculogenesis. Increased expression is also associated with myeloid cell recruitment, cancer cell intravasation and reduced patient survival.	[87, 95, 138, 159, 160, 168, 169]
MMP11	Increased expression associated with faster cancer progression and decreased patient survival.	[170–172]
MMP12	Increased expression associated with impaired cancer progression and improved survival in patients.	[83, 173]
MMP14	Numerous roles including the regulation of epithelial cell migration, vascular stability and regulating cancer cell migration and invasive potential.	[58, 100, 174, 175]
**TIMPs**		
TIMP1	Demonstrated to have both anti-tumor and pro-tumor functions. Anti-tumor functions include the suppression of tumor growth and angiogenesis. Pro-tumor functions include the promotion of tumor growth, recruitment of cancer associated fibroblasts and the promotion of pre-metastatic niche formation. Expression levels also correlate with poor patient survival.	[110–112, 176, 177]
TIMP2	Demonstrated to have both anti-tumor and pro-tumor functions. Anti-tumor functions include the impairment of tumor angiogenesis and invasion. Pro-tumor functions include the promotion of apoptosis resistance in tumor cells and promotion of metastatic spread.	[113, 178, 179]
**LOX Family**		
LOX	Numerous roles in supporting the metastatic cascade, including the promotion of an invasive phenotype in cancer cells, formation of the pre-metastatic niche and increased metastatic spread. Known to increase the stiffness of the ECM and to support cancer progression.	[44, 64, 115, 116, 141, 180]
LOXL2	Increased expression associated with increased invasive potential, metastatic spread and poor patient outcome. Also shown to be essential for developing a microenvironment supportive of cancer progression.	[116, 118, 151, 181–183]
LOXL4	Upregulated in cancer cells and linked to increased metastatic potential.	[184–187]
**uPA**		
uPA	Shown to have an important role in promoting the metastatic potential of cancer cells through increased tumor angiogenesis and cancer cell intravasation. Expression also associated with decreased patient survival.	[87, 120, 188, 189]
**ADAMs**		
ADAM8	Upregulated in cancer cells and linked to increased invasive behavior and reduced patient survival.	[129, 190]
ADAM9	Important roles in supporting tumorigenesis and the generation of poorly differentiated tumors. Expression is also associated with increased invasive and metastatic potential of cancer cells, and reduced patient survival.	[125, 127, 128, 191, 192]
ADAM10	Shown to regulate E-cadherin function, promote carcinogenesis, cancer cell proliferation and protection against apoptosis.	[124, 193, 194]
ADAM12	Known to support cancer progression by promoting cancer cell growth and conferring resistance to apoptosis.	[126, 195, 196]
ADAM15	Upregulated at the invasion front of tumors and correlates with metastatic progression.	[197, 198]
ADAM17	Increased expression associated with increased invasive behavior, faster progression and poorer outcome in patients.	[199–201]

Numerous studies have demonstrated that MMP overexpression is a common feature across various cancers [81–84]. MMP is upregulated by a variety of stromal and cancer cells within the tumor microenvironment [80, 85, 86]. Some of the first evidence for the pro-tumor roles of MMPs came from early MMP ablation studies in animal models. Using a mouse model, Kim et al. demonstrated that MMP9-expressing cancer cells were capable of entering the bloodstream, and their inhibition using the MMP inhibitor marimastat reduced cancer cell intravasation by over 90 % [87]. Consistent with these findings, other studies showed that MMP9 deficient mice developed significantly fewer metastatic colonies compared to wild-type mice following cancer cell inoculation [88]. Perhaps most convincingly, the reduced capacity of MMP9 deficient mice to develop metastatic colonies was reversed with the transplant of MMP9-expressing bone marrow-derived cells [89]. Together, these studies demonstrate that both tumor and stromal-derived MMP9 are necessary for successful metastatic tumor development. Further evidence supporting the importance of MMP function in metastatic cancer progression comes from clinical studies, showing elevated MMP expression correlating with poor prognosis in almost all forms of cancer [84]. For example, the expression of MMP9, MMP13 and MMP14 all correlated with poor survival in patients with breast cancer [90–92], and expression of MMP1 and MMP2 was associated with highly aggressive breast cancer that metastasizes rapidly to the lung [93].

Recent studies have begun to elucidate how MMPs support metastatic cancer development [78, 79] and it is becoming increasingly clear that MMPs have pleiotropic roles and effects on both tumor and stromal cells. For example, MMP2 and MMP9 were upregulated in pre-cancerous nodules where they promoted a switch to an angiogenic phenotype, resulting in tumor transformation and growth [94, 95]. Correspondingly, inhibition of MMP2 and MMP9 using the SB-3CT inhibitor reduced the incidence of liver metastasis and increased the survival of mice with T cell lymphoma [96]. Another interesting insight into MMP function within tumors was reported by Sternlicht et al. who found that induction of MMP3 expression in mammary epithelial cells facilitated the formation of mesenchymal-like tumors [97]. This epithelial to mesenchymal transition of cancer cells is an early phenotypic change associated with aggressive cancer development [98]. However, the tumors grew independent of MMP3 expression after initial formation, suggesting that MMP3 is important during initial tumor development. In support of this, MMP3 overexpressing transgenic mice developed spontaneous pre-malignant lesions and mammary cancers despite the lack of carcinogens or pre-existing gene mutations, indicating that abnormal MMP3 activity is sufficient to initiate cancer cell transformation [97].

MMPs also cleave certain ECM components to expose functional cryptic sites that may be pro-tumorigenic [99]. MMP2 and MMP14 have been shown to cleave the laminin-5 γ2 chain to expose cryptic sites capable of inducing epithelial cell migration [100]. Petitclerc et al. demonstrated that upon cleavage, collagen acts as a ligand for the α_V_β_3_ integrin expressed on malignant melanoma cells and the resultant interaction promoted the survival and growth of melanoma cells [101]. Furthermore, other studies found that cleavage of collagen IV by MMPs exposed a cryptic site that promoted angiogenesis and tumor growth in CS1 melanoma and HT1080 human fibrosarcoma cells [102]. Taken together, MMPs have multiple roles in supporting tumor development and progression, including facilitating initial tumor formation, promoting tumor angiogenesis and cancer cell motility.

Although the majority of MMPs appear to have pro-tumorigenic functions within the tumor microenvironment, it is important to note that some MMPs may have anti-tumor roles. In a mouse model of chemical carcinogenesis, the incidence of skin papillomas and fibrosarcomas was greater in MMP8 deficient mice compared to wild-type, and could be reversed by transplanting MMP8-expressing hematopoietic cells, suggesting that MMP8 has protective effects against cancer transformation and growth [103]. Similar protective roles have been reported for MMP3; Witty et al. demonstrated that MMP3 overexpression reduced the incidence of chemical-induced skin carcinomas following carcinogen treatment [104]. Interestingly, Takeha et al. showed that the number of MMP9-expressing macrophages along the infiltrating margin of tumors was inversely associated with the incidence of liver metastasis and an infiltrating growth pattern in colorectal cancer [105]. This suggests that MMP9 may also have protective effects in contrast to its role in promoting cancer cell transformation and initial tumor development. In support of an anti-tumor role for MMPs, treatment with the MMP inhibitor tanomastat, which has selective activity against MMP2, MMP3 and MMP9, was shown to lead to a worse outcome compared to standard treatments [106]. Collectively, these results indicate that certain MMPs may have additional anti-tumor functions although the exact mechanisms of MMP-mediated anti-tumor effects are still unclear. One possible mechanism may depend on the ability of MMPs to generate the angiogenesis inhibitors angiostatin and endostatin within tumors, which can inhibit the proliferation of endothelial cells and prevent the “angiogenic switch” required for efficient tumor development [107, 108].

It is clear that MMPs have far more complex roles within the tumor than first thought. Numerous studies have now demonstrated that MMPs are able to both support and inhibit primary tumor development and subsequent metastasis. In some instances, such as in the case of MMP9, the same enzyme is able to produce opposite effects. This discrepancy may be due to differences in function and expression of different MMP types, and has important implications for the use of MMP inhibitors to treat metastatic cancer, since broad spectrum inhibition of MMPs will ablate both anti-tumor and pro-tumor functions. Further studies are needed to determine which MMPs are polarized towards supporting metastatic cancer progression and what factors determine the tumor-promoting functions of MMPs.

### Tissue Inhibitor of Metalloproteinases (TIMPs)

Although MMPs clearly play an essential role in cancer metastasis, other groups of ECM regulators including the tissue inhibitor of metalloproteinases (TIMPs) also have important influences on metastatic progression [109]. Thus far, four members of TIMPs have been identified, each containing an N terminal domain that slots into the active site of MMPs, analogous to an MMP substrate, which then inhibits MMP activity. Each TIMP family member may have selective inhibitory properties. For example, TIMP2 and TIMP3 are particularly effective at inhibiting membrane-bound MMPs. TIMPs have been shown to influence metastatic cancer progression, mainly through inhibiting MMP activity. Buck et al. demonstrated that high levels of circulating TIMP1 impaired chemical-induced carcinogenesis and consequently, cancer progression [110]. Consistent with this, Ikenaka et al. used a TIMP1 transgenic mouse model to demonstrate that high TIMP1 expression suppressed tumor growth and angiogenesis [111]. These results demonstrate protective functions of TIMPs against cancer progression. However, similar to MMPs, TIMPs have also been shown to mediate pro-tumor functions. High stromal levels of TIMP1 in human cancers were found to promote cancer growth, promote the recruitment of cancer associated fibroblasts and accelerate cancer progression [112]. This is in keeping with elevated TIMP1 and TIMP2 expression in human cancers and their correlation with poorer prognosis [84, 113]. Hence, similar to the MMPs, TIMPs have opposing effects in cancer progression and further work is required to elucidate the factors and mechanisms influencing TIMP function within the tumor microenvironment.

### Lysyl Oxidase (LOX)

The lysyl oxidase (LOX) family is another group of ECM regulating enzymes with important influences on ECM biodynamics. The LOX family currently consists of 5 members of copper-dependent amine oxidases that are expressed by stromal cells in pre-malignant tissues. LOXs mediate cross-linking between collagens and elastin to increase ECM stiffness and consequently, its capacity to support tumor growth and invasion [51, 64, 114]. LOXs are overexpressed in a variety of cancers and are shown to be markers of increased metastasis, progression and reduced patient survival [64, 115]. Kirschmann et al. observed LOX expression in breast cancer cells with highly malignant phenotypes and inhibition of its function significantly decreased invasive capacity, suggesting a role in tumor cell invasion [116]. Peinado et al. demonstrated that LOXL2 and LOXL3 interacted with SNAI1 to induce epithelial to mesenchymal transition, and knockdown of LOXL2 alone decreased tumor growth and reduced invasive and angiogenic markers within the tumors [117]. In keeping with this finding, Barker et al. showed that LOXL2 promoted metastatic spread through the upregulation of MMP9 and TIMP1 expression [118]. LOX-mediated cross-linking of the ECM has also been shown to promote tumor progression by enhancing integrin signaling, which can inhibit cancer cell apoptosis, regulate cancer stem cell function and growth factor signaling [44]. Taken together, these results indicate that LOX proteins have multiple pro-tumorigenic effects and suggest that their inhibition could be a potential strategy to impair cancer progression and improve patient outcome.

### Urokinase (uPA)

Similar to the LOX proteins, urokinase (uPA) has also been shown to have key functions in tumor progression [119]. uPA is a serine protease forming a critical component of the urokinase-type plasminogen activator system and responsible for the generation of plasmin, an enzyme capable of degrading most ECM proteins. The importance of uPA is highlighted in studies demonstrating its association as a prognostic marker in patients with cancer. Weigelt et al. found that higher levels of uPA activity correlated with a more aggressive phenotype in metastatic breast cancer [120]. Similarly, Shiomi et al. demonstrated that elevated expression of uPA predicted the invasive behavior of esophageal cancer and also correlated with patient survival [121]. In support of uPA’s role in tumor progression, Kim et al. demonstrated that in the absence of surface uPA, cancer cells were incapable of intravasation, despite high expression levels of MMP9 [87]. A possible cooperation between uPA and MMP9 is reflected in the dependence of plasmin, generated by uPA, for the activation of latent MMPs within tissues. This is an exciting prospect, as it suggests that the pharmacological inhibition of uPA may suppress pro-tumor functions of both uPA and certain MMPs such as MMP9. Equally however, the uncertainties surrounding the functions of MMP9 within the tumor microenvironment mean that inhibition of uPA may instead support cancer progression.

### A Disintegrin and Metalloproteinase (ADAM) family

Another group of ECM-regulating enzymes important in metastatic cancer progression is the ADAM proteins, a disintegrin and metalloproteinase family of transmembrane and secreted enzymes [122, 123]. These enzymes are closely related to the MMPs and serve a wide range of functions including cell migration, cell fate determination and regulation of immune responses. Although all ADAMs contain a metalloproteinase domain, only 13 of the 21 members expressed in humans exhibit proteolytic activity on the ECM, suggesting that the remaining 8 may have alternate functions aside from proteolysis. Some members of the ADAM family have been implicated in metastatic cancer progression. Maretzky et al. showed that ADAM10, similar to MMP3, was able to induce epithelial to mesenchymal transition of tumor cells through the cleavage of E-cadherin [124]. These results suggest that ADAM10 may be important for initial tumor development and the acquisition of aggressive metastatic behavior. Mazzocca et al. demonstrated that a secreted form of ADAM9 was able to promote cancer cell invasion by binding the α6β4 and α2β1 integrins [125]. ADAM12 has also been shown to protect against breast cancer cell apoptosis [126]. Overall, these findings indicate that certain ADAM proteins are important for metastatic initiation and progression. In support of these experimental studies, clinical data demonstrated that the expression of ADAM8 and ADAM9 associated with increased metastatic spread and reduced patient survival in a range of cancers [127–129]. Hence, certain members of the ADAM family may represent attractive targets in impairing metastatic cancer progression.

## Myeloid Cells and Their Relationship with the ECM-Regulating Enzymes

**Fig. 2:**
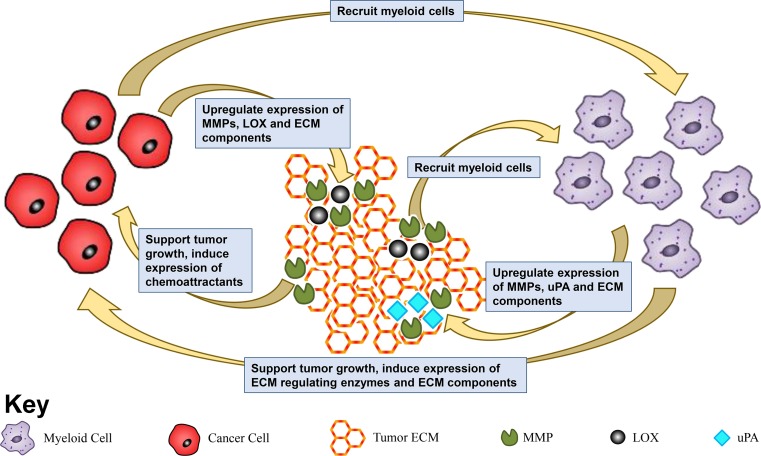
The cooperative relationship between myeloid and cancer cells, and the ECM in support of cancer progression*.* Myeloid and cancer cells produce ECM regulating enzymes such as MMPs, LOX and uPA to alter the tumor ECM. In turn, the tumor ECM mediates function of the myeloid and cancer cells, creating a complex and interdependent relationship that favors cancer progression and metastatic development

Tumor-associated myeloid cells are essential for efficient primary tumor development and metastatic spread. However, very little is known about how myeloid cells are able to promote metastatic progression. Given that efficient metastatic spread and growth is heavily reliant on the tumor ECM, it is likely that myeloid cells mediate the biodynamics and functions of the ECM within the tumor microenvironment as a potential mechanism to promote cancer development. In turn, tumor ECM may be able to influence the functions of infiltrating myeloid cells. In the following sections, we discuss a growing body of evidence supporting a reciprocal relationship between myeloid cells and the tumor ECM in supporting metastatic cancer progression; these interactions are summarized in Fig. 2.

Myeloid cells may regulate ECM function and the consequent effects on malignant progression via direct production of ECM regulating enzymes. Infiltrating myeloid cells express MMPs, and whilst cancer cells and other stromal cells also contribute to MMP expression within the tumor microenvironment, myeloid cells are the predominant source of MMPs in a range of invasive cancers including breast, bladder and ovarian carcinomas [130–132]. Using a transgenic mouse model of skin cancer, Coussens et al. showed that transplantation of MMP9-expressing hematopoietic cells can reverse the impaired development of metastatic cancer in MMP9 null mice [89]. Hence, MMP9 expression by infiltrating hematopoietic cells is sufficient to instigate metastatic growth. Additionally, primary tumors induced MMP9 expression in lung macrophages, which consequently promoted lung metastasis [133]. Ardi et al. also demonstrated that MMP9 expressed by neutrophils may be more readily activated to stimulate angiogenesis [134]. Altogether, these studies demonstrate the importance of MMPs expressed by infiltrating myeloid cells for cancer progression, and suggest that inhibition of myeloid cell recruitment, or inhibition of myeloid cell-derived MMP may inhibit cancer metastasis. Similar to the MMPs, uPA is also predominantly synthesized by tumor-associated macrophages in a number of different cancers [135, 136], and increased uPA expression in tumor-associated macrophages correlated with relapse incidence and decreased survival in patients with breast carcinomas [137].

Whilst myeloid cells may express MMPs to promote malignant progression, MMPs themselves can influence myeloid cell function, suggesting a reciprocal relationship. MMP7 and MMP9 induced syndecan 1 and CXCL6 production in tumor cells, which act as chemoattractants for neutrophils and mediate their influx to the tumor microenvironment [138, 139]. Similarly, MMP3 has also been shown to function as a chemoattractant for macrophages [140]. These studies suggest a positive feedback loop between MMP expression and myeloid cell recruitment, where the expression of MMPs by myeloid cells may stimulate additional recruitment and ultimately, increase the efficiency of metastatic cancer progression. Similarly, LOX proteins expressed by cancer cells accumulate at potential metastatic sites, where they mediate collagen IV crosslinking, which in turn, triggers the recruitment of hematopoietic cells to form the pre-metastatic niche [141].

Although myeloid cell-derived expression of ECM regulating enzymes is important in supporting tumor progression, it is likely that myeloid cells employ other mechanisms to contribute to the deregulated ECM dynamics observed within tumors. In keeping with this, we recently found that depletion of CD11b^+^ myeloid cells in a mouse model of colorectal cancer liver metastasis significantly decreased expression of collagen and laminin isoforms by cancer cells, suggesting that myeloid cells may regulate expression and deposition of certain ECM components via effects on cancer cells [142]. However, we cannot exclude the possibility that myeloid cells themselves can produce and deposit additional ECM components in the same setting. Evidence in support of this comes from studies on Kupffer cells, the main population of myeloid cells within the liver. Kupffer cells are known to have important anti-tumor functions, with numerous studies having demonstrated their ability to clear circulating and dormant metastatic cells residing in the liver, thus reducing the incidence of liver metastasis [143]. However, Kupffer cells also have pro-tumorigenic effects during liver metastasis [143, 144]. Kupffer cells are known to produce a number of different ECM components [145], which may contribute to their ability to assist metastatic colonization of the liver. Another possibility to consider is that infiltrating myeloid cells may induce abnormal ECM expression and deposition by surrounding stromal cells such as cancer-associated fibroblasts, which have been shown to produce high levels of ECM molecules within tumors [50].

The increased ECM deposition induced by myeloid cells may have many important implications. As mentioned earlier, myeloid cells are recruited to sites of metastasis spread or pre-metastatic sites and are essential for efficient metastatic foci expansion and development [37–39]. Given the importance of a deregulated ECM for cancer progression, it is likely that myeloid cells contribute to abnormal ECM expression and structure to form a suitable pre-metastatic niche and/or a hospitable tumor microenvironment as illustrated in Fig. 1b. Many cancer cells implant at secondary sites only to remain dormant for years and fail to establish macrometastases [146]. Myeloid cell recruitment and consequent distortion of ECM composition and dynamics could be a mechanism to transform an otherwise dormant tumor into one capable of forming macrometastases.

## Inhibition of Tumor ECM for Therapeutic Treatments

To date, there has been little progress in developing therapeutic agents to reverse or prevent changes that occur in the ECM during cancer progression. Given the evidence supporting a key role for MMPs in promoting metastatic cancer development, these ECM regulating enzymes were considered attractive therapeutic targets. However, numerous MMP inhibitors including marimastat and tanomastat have performed disappointingly in clinical trials [106, 147, 148]. Patients showed no change in overall survival when administered these MMP inhibitors. However, it is too early to conclude that MMPs are not suitable targets and there are a number of reasons that could explain their ineffectiveness. One major concern was whether the doses used were high enough to inhibit MMP activity [106]. It was proposed that patients who developed musculoskeletal symptoms were the only ones to have received a dose high enough to inhibit MMP activity. King et al. found that patients who were given the MMP inhibitor marimastat developed musculoskeletal side effects but had a significantly higher survival time compared to patients who were given marimastat but developed no side effects [149]. Another important argument is that MMP inhibitors were tested in cases when metastatic spread was too far advanced to show any therapeutic benefit. The findings that MMPs may have important roles in promoting early tumor initiation and development [97] implied that MMP inhibition will be far more successful in patients with early metastatic disease. A further criticism is that previous MMP inhibitor trials did not account for the fact that certain MMPs are clearly more important in supporting cancer progression compared to others. The majority of MMP inhibitor agents used had non-specific, broad spectrum activity against a variety of different MMPs. From the experimental studies discussed in this review, it is clear that MMP3 and MMP9 play particularly important roles in supporting metastatic cancer progression and may account for the greater part of MMP-mediated pro-tumor functions. Selective inhibition of MMP3 and MMP9 may minimize the inhibition of anti-tumor functions amongst other MMPs. More promising results from MMP inhibition clinical trials may be achieved by ensuring an adequate dosage for complete MMP inhibition, as well as selectively inhibiting MMPs with pro-tumor functions.

Numerous studies have also demonstrated that the inhibition of LOX has the capacity to inhibit tumor progression and metastasis [115, 141, 150]. Indeed, administration of a LOXL2 inhibitory monoclonal antibody impaired tumor growth and metastatic colonization in a mouse model through effects on the tumor microenvironment [151]. Despite this, there has been relatively little attention dedicated to developing inhibitory therapeutic agents against LOX. So far, these agents include competitive inhibitors such as βAPN [115, 151] and neutralizing antibodies such as simtuzumab. Unfortunately, simtuzumab was ineffective in Phase 2 clinical trials as its administration produced no benefit in patients with pancreatic cancer [150]. However, this disappointing result may be due to the selective inhibition of LOXL2 as opposed to broad spectrum inhibition and in addition, may be due to the trial being conducted in patients with advanced cases of pancreatic cancer.

Aside from MMPs and LOX, a variety of other enzymes such as TIMPs, uPA and ADAMs may also be considered potential targets. However, experimental studies on TIMPs indicate that they mediate anti-tumor functions as well, and their inhibition may have unexpected outcomes. Regarding uPA, the potential cooperation between uPA and MMPs suggest that uPA inhibition could also hinder MMP function and thus lead to inhibition of tumor progression. A few uPA inhibitors such as upamostat have already entered clinical trials [152, 153] and whilst the initial results appeared promising, further trials are needed before the efficacy of uPA as a therapeutic target can be concluded.

With the collective evidence supporting the cooperative relationship between myeloid cells and the tumor ECM, it seems likely that ablation of myeloid cells or inhibition of their function may inadvertently affect the ECM. However, myeloid cells perform a wide range of physiological functions thus targeting them for the treatment of metastatic cancer may represent a significant challenge. The myeloid lineage of hematopoietic cells is an indispensable part of the innate immune system, meaning that ablation of their function is likely to result in immunosuppression. As well as providing defense against infectious pathogens, myeloid cells are known to have important anti-tumor functions, and their targeting may instead promote cancer progression by suppressing myeloid-cell mediated cancer cell destruction. As a result, a more specific approach may be to disrupt myeloid cell-ECM interactions and impair their cooperative relationship.

## Conclusion

The importance of infiltrating myeloid cells and the tumor ECM for metastatic cancer progression is now widely appreciated but we are only just beginning to understand how these extrinsic factors interact with each other within the tumor microenvironment. Numerous studies have now demonstrated the ability of myeloid cells to distort the normal functions of ECM regulating enzymes, as well as mediating the pathological deposition of pro-tumor ECM components within the tumor microenvironment. In turn, certain components of the ECM can regulate the behavior and function of myeloid cells, and this cooperative interaction appears to be a necessary factor in metastatic cancer progression.

However, it has been clear from early attempts that targeting this cooperative relationship between myeloid cells and the tumor ECM is fraught with challenges. The range of potential therapeutic targets identified is vast and many previous attempts at inhibiting key players in this relationship have failed. MMP expression by myeloid cells in the tumor microenvironment has been shown to be important for tumor progression. However, it is difficult to target MMPs specifically expressed by myeloid cells and non-specific targeting of MMPs have not led to significant inhibition of tumor progression. Additionally, inhibition of the LOX family, whilst initially promising in experimental studies, has produced disappointing results in clinic. An alternative approach to restoring normal ECM enzyme function may be to prevent the initial recruitment of myeloid cells to the tumor microenvironment. This would additionally serve to prevent myeloid cell-mediated deposition of pro-tumor ECM components and impair another important source of support for cancer progression. However, this may be difficult to achieve without disrupting myeloid cell function in patients altogether.

Despite our limited success in clinical trials, we remain positive that the restoration of normal ECM biodynamics within tumors will have significant impact on metastatic cancer progression. Given the evidence collected over the years, a larger focus needs be directed at means of impairing the cooperative relationship between myeloid cells and the tumor ECM. As well as additional efforts directed at inhibiting MMP and LOX, other ECM regulating enzymes such as uPA need to be considered and investigated further. Furthermore, an important avenue of work to pursue is to identify other potential mechanisms by which infiltrating myeloid cells influence tumor ECM. This will improve our understanding of the relationship between myeloid cells and the tumor ECM, as well as providing additional therapeutic targets for treatment of metastatic cancers.

## Abbreviations

ADAM: A disintegrin and metalloproteinase
CCL: chemokine (C-C motif) ligand
CXCL: chemokine (C-X-C motif) ligand
CD: cluster of differentiation
CSF: colony stimulating factor
EGF: epidermal growth factor
ECM: extracellular matrix
FGF: fibroblast growth factor
IL: interleukin
LOX: lysyl oxidase
M-CSF: macrophage colony-stimulating factor
MMP: matrix metalloproteinase
MDSCs: myeloid-derived suppressor cells
PI3 kinase: phosphoinositide 3-kinase
SNAI1: Snail family zinc finger 1
TIMP: tissue inhibitor of metalloproteinase
TGFβ: transforming growth factor β
TAMs: tumor associated macrophages
TANs: tumor associated neutrophils
TNF: tumor necrosis factor
uPA: urokinase
VEGF: vascular endothelial growth factor
βAPN: β-aminopropionitrile
